# Poliomyelitis Anterior Acuta (Acute Inflammation of the Anterior Gray Horns, Acute Atrophic Spinal Paralysis)

**Published:** 1892-12

**Authors:** 


### Poliomyelitis Anterior Acuta (Kussmaul) (Acute Inflammation of the Anterior Gray Horns, Acute Atrophic Spinal Paralylis).

*Etiology.*—This affection is most frequently met with in childhood, the disease appearing between the ages of six months and three years in thirty-two out of thirty-four cases observed by West. It may, however, occur at so early an age as ten days, and a disease essentially the same occurs in the adult. Heine asserts that the disease occurs in the healthiest children, and that neither sex nor hereditary predisposition appears to exercise any influence on its causation. It appears, according to Drs. Barlow and Wharton Sinkler, to be more common during the summer than the winter months.

Injuries of various kinds are often said to be the cause of the disease, and nurses are frequently blamed unjustly by parents who, unable to believe that such a striking phenomenon as paralysis can occur suddenly without appreciable cause, imagine that the child has been lamed by a fall through the carelessness of its attendant. The most usual exciting causes are painful dentition, and exposure to cold, more especially when the body is overheated, and the affection often occurs in children, and occasionally in the adult, during or soon after an attack of measles, scarlatina, small-pox, typhus, and other acute affections.

*Symptoms.*—Although this disease is essentially the same in children as in adults, yet the symptoms differ so much in the two as to demand separate description. The disease will be first described as it occurs in children.

#### *a.* Infantile Spinal Atrophic Paralysis.

It will conduce to clearness if, like Laborde, we divide the clinical history of this affection into the periods of (1) invasion;

---

*Since writing the above, four more cases of Infantile Spinal Paralysis have come into my hands, two of them *neglected cases.*

(2) remission; (3) regression of paralytic phenomena; (4) atrophy with deformities. It must, however, be remembered that these periods overlap, and that this sub-division is merely adopted for the sake of convenience.

(1) *The Period of Invasion.*—The disease is commonly ushered in by a more or less intense fever, which is often preceded by general malaise, pain in the head or in the back, mental irritability, fretfulness, and startings of the limbs. As a rule, the fever is of short duration, lasting only from one to two days. In some cases it passes off in a few hours, while in others it continues from fourteen to sixteen days, or even longer. As the fever increases the cerebral symptoms become more pronounced, confusion of ideas and slight somnolency are observed, and the child may become unconscious, or delirium of varying degrees of intensity may supervene. The disease was ushered in by convulsions in thirty out of seventy cases collected by Duchenne, these convulsions being, most probably, the ordinary eclamptic attacks which so frequently precede every acute febrile disease in childhood. All general symptoms are sometimes absent; the child is put to bed apparently in good health, and is found paralyzed in the morning.

(2) *The Period of Remission.*—After the initial symptoms have subsided it is observed that the patient is unable to move one or more of his limbs, or the paralysis may not attract attention until the child is taken out of bed for some purpose, and then the relaxed and helpless condition of the affected extremities can hardly fail to be noticed. The paralysis is probably never fully developed at once; it increases gradually and reaches its maximum extent and degree in the course of a few hours, in a day or two, and occasionally not until a longer period has elapsed. Relapses sometimes occur. After a first attack one limb is paralyzed, and a few days afterward, during which rapid improvement has been taking place, the child is seized with a second febrile attack and other limbs become paralyzed. A case is reported by Laborde in which the paralysis did not become permanently established until the third attack.

The *distribution* of the paralysis is very variable. It often involves not only the four extremities, but also the muscles of the vertebral column, and even the intercostal muscles and those of the neck are not always spared. Sometimes the lower extremities alone are paralyzed, but the upper extremities are seldom exclusively affected. The paralysis assumes in some cases the form of a hemiplegia, and in these the side of the neck, of the face, and of the tongue may be implicated at first, and may on rare occasions remain permanently paralyzed.

*Sensory disorders* are almost entirely absent during the whole course of the disease. At the outset patients may complain of pains and various paræsthesia, but these symptoms are of short duration. A certain degree of cutaneous hyperæsthesia or rather hyperalgesia has been described as being present during the febrile stage; but this tenderness to touch probably depends upon affections of deeper structures, such as rheumatic inflammation of joints. The cutaneous sensibility is sometimes blunted in the paralyzed extremities in old standing cases; but this probably depends upon underlying nutritive and vascular changes.

*Reflex action* is completely lost in all the muscles which are severely paralyzed, and it is much lowered or temporarily extinguished in the muscles which are slightly affected.

The *tendon reactions* are also absent in the paralyzed muscles.

The *functions of the bladder and rectum* are rarely affected. During the first days there may be retention, but more frequently there is incontinence, and the stools may be passed involuntarily. These disorders disappear in from three to eight days from the commencement, except in young children, in whom a slight incontinence of urine may remain for some time.

(3) *The Period of Regression.*—After a certain time, which varies from a few days to a few weeks, some of the paralyzed muscles begin to improve, and in a few cases a complete recovery takes place. The cases which recover have been described by Kennedy under the name of *temporary spinal paralysis.* But, as a rule, only some of the muscles are completely restored, while the rest remain permanently paralyzed. When the paralysis is general it often happens that the upper half of the body is the first to show signs of amendment, the paralysis disappearing rapidly from the neck, upper extremities, and trunk, and becoming restricted to the lower extremities. This improvement, which Laborde calls the period of *first regression*, is followed after a variable interval of time by a second period of amendment, which the same author calls the period of *second regression.* During the second regression some of the muscles of the lower extremities undergo a progressive improvement, and one limb may be restored to full motive power, while one or more of the muscular groups of the other limb may remain permanently affected, particularly in the anterior and external group of the leg. In some cases improvement takes place from below upward instead of from above downward, and then the paralysis becomes permanently localized in a superior extremity, or occasionally in the trunk or neck. The permanently paralyzed muscles are implicated in groups according as they are associated in their action, and not in accordance with the peripheral distribution of their motor nerves.

(4) *The Period of Atrophy and Deformities.*—All the muscles which are severely paralyzed become the subjects of a *rapidly progressive atrophy*, and even those which are but slightly affected waste to some extent, but soon recover. The atrophy becomes well marked in the course of a few weeks in the muscles which are severely affected, and after a time they become so attenuated that the bones seem to underlie the skin. Sometimes the muscular atrophy is masked by the accumulation of fat in the connective tissue, and consequently the loss of volume is not always a trustworthy test of the degree to which the muscle has become atrophied.

The *electrical reactions* of the affected nerves and muscles may vary from a simple diminution of the normal reactions to the *partial* or the *complete* reaction of degeneration. It was first shown by Duchenne that the Faradic irritability of both nerves and muscles sinks quickly in those which are severely attacked, and becomes lost on the seventh day or during the course of the second week. He laid it down as a rule that all the paralyzed muscle in which the Faradic irritability is only more or less diminished during the course of the second week do not remain permanently paralyzed, and that the restoration is the more prompt and complete the less the Faradic irritability is diminished.

*Arrest of development of the osseous system* often occurs, and it may be altogether out of proportion to the degree in which the muscles are paralyzed. The greater part of the muscles of a limb may, indeed, be lost while the bones are almost entirely unaffected, and, conversely, a limb may be considerably shortened while only one or two muscles are atrophied. The paralyzed lower extremity may be found from two to six inches shorter than the corresponding healthy limb, and the upper extremity may be similarly affected, although not generally to the same degree. The long bones are thinner than normal; they are porous, friable, and yielding; their epiphyses and processes grow smaller and less distinct; the paralyzed hand or foot is shorter, narrower, and thinner than the sound one; and even the pelvis may be arrested in its development.

The *joints* become unsually movable, partly from disappearance of the articular extremities of the bones and partly from relaxation and stretching of their ligaments, and occasionally the changes undergone are so great that the patient is able to dislocate a joint without experiencing any discomfort.

The *skin* of the affected extremity is flabby and so inelastic that it retains, for a long time, slight pressure marks, such as that made by the stocking. The surface of the limbs is of a mottled or bluish color, it is remarkably cold to the touch, and in old cases its temperature may be from 5° to 12° F. lower than that of the corresponding healthy limb. The skin is liable to chilblains, and indolent ulcers form on slight provocation, these nutritive changes being accompanied, and probably caused by a diminution in the calibre of the arteries.

The *deformities* occurring in the affected limbs give to this disease some of its most characteristic features. Some pathologists believe that the deformities are produced by the predominant action of the healthy muscles, the normal *tonus* being destroyed in the affected muscles; but Volkmann asserts that the deformity is produced mainly by the weight of the limb itself, because the position generally assumed by the foot is only a higher degree of that which it assumes when unsupported and left free from the action of the muscles. The influence of gravity on the position of the limb ought certainly not to be ignored, but the position assumed by the foot in talipes calcaneus when the muscles of the calf are paralyzed shows that gravity is not the only, perhaps not the chief force which is operative in the production of these deformities. Two other factors, at least, must be taken into account. The first is that the paralyzed muscles often permit the limb to assume a position in which the ends of their healthy antagonists are more or less permanently approximated, and the latter consequently become permanently shortened by undergoing "adapted atrophy." The second factor is that the paralyzed muscles themselves may become permanently shortened, either from arrested development or from proliferation and subsequent retraction of their connective tissues. Of all the deformities which occur in infantile paralysis, talipes equinus and equino-varus are the most frequent, because the muscles most frequently paralyzed are the long extensors of the toes, the tibialis anticus, the extensors of the great toes, and the peronei muscles. When the anterior group and the abductors of the foot are affected at the same time talipes equino-valgus, and when the muscles of the calf alone are affected talipes calcaneus is produced, but this form is exceedingly rare, and simple talipes varus is of still rarer occurrence. Another common deformity is the "pes cavus"—"talus pied creux" of the French, in which the sole is hollowed and the instep is rendered prominent. Duchenne thinks it is caused by a more or less complete paralysis of the muscles of the calf, along with simultaneous contraction of the flexors of the foot either the long flexors of the toes or the long peroneus. The great laxity of the ligaments allows the foot to become bent upon itself from the transverse tarsal joint when the foot is unsupported, but when it is placed upon the ground it assumes the form of "flat-foot."

Various deformities occur in the inferior extremity, according to the extent and localization of the paralysis. The anterior and internal muscles of the thigh are those most usually affected above the knee, and then the predominant action of the flexors of the leg on the thigh maintains the leg in a permanent condition of partial flexion (*genu recurvatum*), while the thigh is likewise abducted. This deformity is always associated with flexion of the thigh of the body, and talipes equino-varus. All the muscles of both legs are sometimes paralyzed, so that the patient is compelled to walk on his knees, dragging his small thin legs after him. In still more aggravated cases the muscles of both legs and thighs are permanently paralyzed, so that the small, flexible limbs dangle about like the limbs of a doll (*jambe de polichinelle*). Curvatures of the vertebral column generally result in infantile paralysis from the attitudes imposed by other deformities, but occasionally the curvature is caused more or less directly by the paralysis. Of the direct curvatures, *lordosis*, is the most frequent and important; it is caused by partial paralysis of the sacro-spinal muscles, and in order to prevent the permanent bending forward of the body, by the predominant action of the flexors, the patient involuntarily throws the trunk backward, so that the weight of the trunk is borne by the flexors, while the tension is taken off the partially paralyzed extensors. In this form of lordosis the pelvis is pushed forward, and the buttocks become less prominent than in health.

The deformities of the upper extremities are much less frequent and serious than those of the lower extremities. The muscles of the shoulder, and particularly the deltoid, are the most usual subjects of paralysis and atrophy in the upper extremity. In the severer forms of paralysis of the muscles about the shoulder joint the humerus becomes separated from the glenoid cavity, so that a dislocation is readily produced or may occur spontaneously, the arm hangs powerless by the side, and, to use the apt comparison of Heine, dangles about like the loose end of a flail. The distortions of the forearm and hand are not so frequent or important as to require description.

The general health of the patient is not interfered with in this disease; the organic functions are well performed and the patient may live to extreme old age.

### Treatment.

The treatment of these cases by the allopathic school is of the usual plan. First, every effort is made to reduce the fever with laxative and cool baths.

Then comes the long narrow blister down the spine, or stimulating linaments containing Croton oil, or Turpentine. Internally to assist in overcoming the spinal conjestion Ergot and Iodide of Potassium are given. After all this depleting and exhausting treatment, comes as a matter of course the building-up process, Iron, Phosphoric Acid, Strychnia, and Quinine. Electricity is highly lauded by some, and great caution in its use advised by others. Massage, and other exercise with the use of proper apparatus.

One authority sums up his remarks with the following: It may readily be surmised that this plan of treatment (orthopædic) is one requiring the utmost patience and persistence, and the most loving persuasion and encouragement, for indeed it must be pursued in face of all apparent failure, for months and years. Of electricity we know less than we should.

The old school use it as they do drugs, in such quantity that more harm than good is the constant result.

A very remarkable and interesting series of results is reported by Keeth, the great London surgeon, in the treatment of fibroid tumors.

We find him adopting the smallest possible dose, some patients receiving but a single short treatment and sent home to await the result, which in almost every case results in stopping the discharges and later atrophy of the growth.

I may use a minimum dose of electricity in both of these cases. The allopaths are very guarded regarding the duration and prognosis of Infantile Paralysis.

### Third Case.—Irritation of the Spine.

The third case to which I wish to call your attention is an aggravated case of spinal irritation in a girl eighteen years of age.

Ten or twelve years ago the family physician, who was hunting for spinal diseases, informed the mother of the dangerous condition of the spine, and from that time to the present she has worn a heavy corset.

Several years ago an abscess, deep in the tissues, was said to be on the point of breaking; but it never did, because there was never one to break.

Before the case was placed in my hands, I was consulted as to what was best to do, her former physician having moved from the city.

An examination showed it to be a most aggravated case of spinal irritation, in a psoric person.

Of course, I advised thorough homœopathic treatment, but they were not quite sure I could be correct in my diagnosis, and took her to three of the orthopædic men, none of whom pleased them.

They all agreed with me, however, in diagnosis. Electricity was decided upon and greatly improved the case for a time. As usual, on the old principle that if a little is good, much is better, they overdid it, and she became much worse, and was brought to me to do as I thought best with.

She could go about the house for an hour or two each day, some days more, some less; but most of the time was spent on or in the bed.

She wore a heavy corset, without which support she suffered great pain all through the back and right side. Being naturally studious and ambitious, she made every effort to take music and other studies, but would collapse entirely after the teachers had finished their half-hour instructions.

Cold damp hands and feet; a clear white complexion; very red lips; face flushed and paled alternately when the least tired; and she sang or complained, when not reading, of which she did a great deal in bed.

The selection of the remedy was not difficult. But to get off the harness and have a light brace or support fitted so that she could get into the air and about the house was a very difficult feat to perform.

It was almost impossible to persuade her mother that there was not serious spinal disease; that there was not "dead" bone which should be cut out and must be cut out before the child could recover. The child's figure was almost perfectly symmetrical, but the anxious parent was sure one side was much larger than the other, etc., etc.

Under Calcarea-carb. and daily massage the general condition was greatly improved. The cold, clammy skin became warm and dry, and one by one other symptoms abated.

It was months before a new brace could be worn with comfort, and not until I gave her Selenium, for the constant distress, aching and burning in the back and right side, that this was accomplished.

The effect upon the aching and burning pains was very marked. The relief very great, but was followed by enormous swelling of the breasts, which lasted several weeks.

It was impossible to touch the girl's spine or back without giving her very great pain until recently.

Calc-carb., Ferrum, Silicea, Selenium, and Puls. are all the remedies she has had. The horrors of spinal disease and a distorted figure are removed from her mind.

She attends church and musical entertainments with scarcely any fatigue, and will soon abandon the corset entirely. If properly treated in the beginning she would have never worn a corset and recovered years ago.

The well-known characteristic conditions of this pathological condition are brief and as follows:

### Spinal Irritation.

*Etiology.*—The female sex predispose to this disease, although men are occasionally affected. The members of neuropathic families are generally attacked, and most of the cases occur between fifteen and thirty years of age.

The exciting causes are emotional disturbances, sexual excesses, exhausting diseases, and everything which weakens the nervous system.

*Symptoms.*—The symptoms begin with headache, sleeplessness, increased nervous irritability, ill-defined pains in the back, neuralgiform pains in the face or extremities, and general feebleness, these symptoms gradually increasing in intensity until the disease is fully developed.

The patient now complains of pain in the back, which is aggravated by exertion, and is situated most frequently between the shoulder blades, or in the back of the neck, and less frequently in the loins. The spinous processes of some of the vertebræ are excessively tender to pressure, and over these processes the surface is found to be very sensitive when a hot sponge or the cathode of a galvanic current is applied. Tenderness of the vertebræ to pressure is, indeed, the most constant and important symptom of spinal irritation, and this sign is rendered all the more valuable from the fact that spinal tenderness is never a prominent symptom of myelitis and other organic diseases of the cord.

The patient complains of various paræsthesiæ and neuralgiform pains in the upper and lower extremities, occiput, face, pelvic region, bladder, genitals, or viscera; the slightest exertion occasions great fatigue and exhaustion; and walking soon becomes impossible owing to the excessive pain caused by it.

The motor symptoms consist of fibrillary twitchings, spasms of some muscles, choreic movements, hiccough, and even permanent contractures in rare cases. Epileptic attacks are said to have been occasionalty observed, but it is more likely that these general convulsions were hysterical seizures. A certain degree of muscular weakness may be present in some cases, but real paralysis has never been observed.

The vaso-motor disorders consist of coldness of the hands and feet, and the patients are apt to turn pale or red on the slightest provocation.

The most common visceral disorders met with are eructations, nausea, vomiting, palpitations, asthmatic breathing, cough, vesical spasm, and polyuria. The patient also complains of noises in the ears, dizziness, muscæ volitantes, and other disorders of vision, sleeplessness, and great mental depression or irritability.

### Fourth Case.

The fourth case is one of hemiplegia resulting from cerebral apoplexia.

This is interesting only that it illustrates the benefit to be derived from exercise as soon as it can be taken in all forms of nervous disease.

The apoplexy was so intense and prolonged that no one expected the man to recover, which he did very slowly.

I was called to see the case, months after the attack, and found him tumbled into a wheel chair and able to be held up between two strong men and moved a very difficult step or two. I had him put at once into Darroch's wheel chair, and with marked advantage.

Very soon he could walk all about the room and soon on the walks of his premises.

In mild, or the most severe cases of chronic rheumatism, this chair is of great use.

### Fifth Case.

One of the most troublesome and distressing cases brought to my attention this year is a terribly aggravated case of lateral curvature of the spine in a young girl of sixteen years.

It began in school and is a case of *simple neglect.* No attention was paid to it for a long time. The mother, a very lovely woman in many ways, would not allow herself to believe that her child needed anything more than a mild suggestion now and then to stand erect. Although a pale and weakly child full of psora, a physician's care was considered unnecessary; in fact, avoided; and although amply able and willing to pay any expense attached to treatment of the case, it was neglected from mere inability on the part of the mother to be made to understand the situation.

It is easy to see how a case of this kind would grow worse and worse almost in spite of all that can be done.

Naturally weakly and listless. Objecting to help which annoys her in the least. Growing each month as she developed into womanhood, the increase of weight adds constantly to the difficulties to be overcome.

The physician cannot be too imperative in his demand to be strictly obeyed in these cases from the very beginning.

This case was for two years under an orthopædic surgeon, but grew constantly worse because she did not have the homœopathic remedy, and because he neglected to see that his orders were obeyed.

Resting on an inclined plane and being rolled daily by a trained masseur is the best treatment of these cases, together with a suitable support, if the case is inclined to be serious.

### Sixth Case.

Erskine Kline Malcolm, born in Nova Scotia, April 26th, 1862, come to the States in November 1889, and became a weaver in a cloth mill in Maynard, Mass. One morning in the latter part of September, 1890, he attempted to lift a cut of heavy cloth, weighing seventy-five to one hundred pounds. The floor was smooth, and as he threw the cloth over his left shoulder, he lost his equilibrium and was falling backward, when, by a sharp struggle, he regained his footing. At the moment of this severe effort he was conscious of a sharp pain, which ran from the left side of the neck to the left ear. This was only momentary, and he continued work all day, merely mentioning at home that he had nearly fallen. He noticed no symptom of any injury for about three months. Then, one morning, he observed a stiff feeling on the left side of the neck on waking, which passed off on rising and moving about, and was attributed to a slight cold. But this sensation recurred each morning, augmenting in severity and lasting longer each day, until the skin on the left side of the neck felt tight all the time. Soon he began to be wakened at from two to four A. M. with pain, sharp and drawing, seeming to elevate the shoulder and pull the head down on it. In the middle of February, 1891, he was obliged to stop working in the mill, for the first time since the accident, and then first sought medical advice. His physician prescribed a liniment on the neck each night, the nature of which the patient did not know, except that it was so hot that on the fifth night, after having received no benefit from it, he used too much and was badly blistered. He discontinued the use of the liniment, and continued to grow worse, until he became delirious with pain, and imagined a man hacking with a knife at the back of his head. He was out of the mill until the first of May, with the exception of one day and a half, when he felt improved. All this time he suffered from severe pains which were only temporarily relieved by hot applications, hops, etc., and finally Morphine. When he was again able to work his neck was stiff and his head bent to his shoulder, but the pain was less. He was out for two months in the summer, and on returning to work was put to a loom as weaver. He had hardly begun this when he began to lose the use of the left arm, and was soon completely paralyzed in that limb. There was still some pain in his head and his left knee was weak. Faradism was tried, but to no purpose. He then went to the Massachusetts General Hospital, where salt water bathing of the affected parts was prescribed. He was also directed to rub with a stiff brush and a remedy, the name of which he forgot, and which he consequently did not use. On December first he again went to the Massachusetts General Hospital, where he saw Drs. Putnam and Carter, and where his trouble was first diagnosed by the latter physician as dislocation of the cervical vertebræ. Galvanism was prescribed for the paralysis, but it grew worse rather than better. The surgeon of the Hospital in examining felt a projecting point of a cervical vertebra by pressing on the right side of the neck beside the larynx. He put on a Thomas collar, and asked him to return in a week. Instead of returning he took the advice of a friend and came to New York to the Hahnemannian Hospital. I saw him and prescribed a Darrach jury-mast, and ordered him to exercise in the wheel crutch. Improvement in his arm and leg was immediate and has continued. All symptoms disappeared when pressure was removed from the injured parts, and I have given him no medicine except one dose of Hypericum. He walks in the park several hours at a time, and has charge of the dispensary building attached to the Hospital.

Can dress and undress himself, and from being a helpless cripple is now a useful man about the Hospital. I hope to permantly cure the poor fellow.

Whether he has a *broken neck* or ruptured cervical muscles I cannot determine, but here again is illustrated the wonderful effect of proper *apparatus*, especially the wheel chair.

### Removal of the Ovaries.

Too much cannot be said against the popular and simple operation of removing the ovarian appendages.

Surgeons, young and old, are rushing into this with blind enthusiasm for two reasons.

First, in the old school, it is almost impossible to cure any difficulty which arises from the pelvic organs.

Their very method of procedure warrants "no cure," and invariably they fan the flame.

If the attending physician is religiously inclined his failures are laid to "Adam's fall;" if he is scientific and has discarded all divine authority, he lays it to the innocent microbe, and proceeds to remove the patient from the reach of the creature.

I will not burden you with the details of a dozen cases that are under my care and observation. That there is an occasional case benefited by this operation there is no question, but they are very few.

They invariably are miserable for from a year to the rest of their mortal life.

One case under my care, operated upon by a famous operator seven years ago, has grown worse every year since the operation, and I can do nothing for her.

Another case operated upon two years ago has required my help ever since the operation and it is hardly well yet. She is a remarkably well-developed young woman, and no more required the operation than the surgeon who performed it. It was an outrage, and so on through the dozen.

Then again there are errors on our side. So confident do we become of the effect of our wonderful remedies and our ability to hit the bull's eye every time that we get more than the laugh on us too often.

We do not kill, but by looking through an open door we can see more than through the keyhole.

A case of complete rupture of the perineum with involuntary movements of the bowels was brought to me this year for an operation.

A most excellent Hahemannian, without any examination, had assured her of a cure with remedies only, but failed. Such assurance and carelessness is a "fatal error."

Two cases of water about the knee have afforded me much satisfaction and are worthy of a moment of our time.

I have advocated to the best of my ability in my paper on "spinal operation" the proper use of supports.

Now I am about to condemn them, at any rate, to discard them in cases where they are unnecessarily used.

Two years ago a gentleman applied to me for lameness in the left knee.

He was on crutches, the joint was carefully supported with cotton and a rubber roller bandage.

On removing the dressing I found a large accumulation ot water and great sensitiveness. He could not bear any weight on the foot. As a homœopathic physician had been treating him there had been no medicated applications.

The remedy was not very evident if we regarded the knee, but the history of his general state for the past thirty years was Sulphur, and that only. So he got it according to our method of administering it, and his recovery was remarkable. Very soon he had no use for crutches and has remained well for over a year, although constantly busy. I removed at once the bandage and deprived him of his crutches.

The second case came to the Hahnemann Hospital and was fortunate enough to fall into my hands.

He had been in one of the allopathic hospitals for a longer time than he could afford—being a painter and dependent upon his daily work to support his family.

His knee was also bandaged with a rubber roller and cotton, and had also been repeatedly painted with Iodine and blistered; but it was growing constantly worse.

I called the attention of a warm friend of mine on the surgical staff to the case and told him I would cure it with the medicated remedy only.

He said it could not be done. The remedy was Apis, and in a week the man begged me to let him go to work. But I kept him a week longer to make the cure permanent.

